# Severe Hemorrhage Following Dental Implant Surgery in an Anticoagulated Elderly Patient: A Case Report

**DOI:** 10.7759/cureus.83879

**Published:** 2025-05-11

**Authors:** Mariana Pinto, João Mendes Abreu, Claudia Queirós Henriques, José Pedro Figueiredo, Ana Corte Real

**Affiliations:** 1 Maxillofacial Service - Head, Neck and Skin Surgery Department, Coimbra Local Health Unit, Coimbra, PRT; 2 Coimbra Hospital and University Centre, Clinical and Academic Centre of Coimbra, Coimbra, PRT; 3 Faculty of Medicine, University of Coimbra, Coimbra, PRT; 4 Stomatology Service - Head, Neck and Skin Surgery Department, Coimbra Local Health Unit, Coimbra, PRT

**Keywords:** anticoagulants, blood transfusion, dental implant, oral surgery, postoperative hemorrhage

## Abstract

Even though dental surgery is an effective method of oral rehabilitation, it is imperative to individualize treatment planning and, especially in patients under anticoagulant therapy, to initiate early intervention in the prevention and management of hemorrhagic complications. An 80-year-old Caucasian female patient presented to the emergency department with severe oral bleeding following maxillary dental extractions and immediate implant placement. The patient was on anticoagulation therapy due to pre-existing medical conditions and had been switched to enoxaparin. However, the patient's weight and renal glomerular filtration rate were not taken into account, thus precipitating the event. Postoperative bleeding, although uncommon, can be a serious complication of oral surgery in patients on anticoagulant therapy. The risk of such complications is influenced by several factors, particularly the type and dosage of anticoagulant used, the patient's overall health, and the extent of surgical trauma. This case highlights the critical role of a multidisciplinary approach in the management of patients on anticoagulation therapy who require invasive treatments.

## Introduction

Dental surgery is a safe and effective method of dental rehabilitation. However, it is imperative to individualize treatment planning and early intervention in the prevention and management of hemorrhagic complications, particularly in patients on anticoagulant therapy [1,2].

Current clinical guidelines recommend continuing anticoagulant therapy in specific patients to prevent thromboembolic events. These require precise and minimally invasive surgical interventions and the use of localized hemostatic measures to minimize bleeding risks. Furthermore, the careful selection of patients plays a critical role in ensuring the safety and success of the procedure [3,4].

Although the event of severe hemorrhagic bleeding in dental implant surgery is uncommon, its potential to result in serious complications must not be overlooked [5]. When contemplating implant placement with simultaneous tooth extraction in patients at high risk of bleeding, such as all-on-four surgery, it is imperative to exercise extreme caution and, when feasible, refrain from such procedures [6,7]. Moreover, in severe and catastrophic cases, excessive blood loss may require transfusion and multidisciplinary emergency intervention [8].

This report presents a case of complications arising from simultaneous dental extraction and implant surgery in an elderly patient and focuses on risk assessment and management. It emphasizes the importance of individualized treatment planning and early intervention in preventing and managing hemorrhagic complications. The findings herein advocate a proactive approach prioritizing patient safety and clinical success in dental care.

## Case presentation

This article reports the case of an 80-year-old Caucasian female patient who presented to the emergency department (ED) with catastrophic oral bleeding following maxillary dental extractions and immediate implant placement.

The patient's medical history included atrial fibrillation, mitral valve replacement, and tricuspid annuloplasty. She received a dual-sensor ventricular demand rate-responsive (VVIR) pacemaker in August 2014 for brady-tachy syndrome. Additional pre-existing comorbidities included arterial hypertension and dyslipidemia. Relevant medication comprised anticoagulation therapy with warfarin, adjusted according to International Normalized Ratio (INR) levels. Furthermore, the patient was prescribed a combination of medications for her comorbid conditions, including furosemide, perindopril, bisoprolol, spironolactone, atorvastatin, pantoprazole, ursodeoxycholic acid, tiotropium bromide and fluticasone with formoterol. No documented allergies were reported.

Prior to surgery, her general practitioner, responding to her dentist's request, changed her anticoagulant therapy from warfarin to enoxaparin at a dose of 60 mg every 12 hours. The patient's weight and renal glomerular filtration rate were not considered.

During the preliminary procedure, the patient experienced atypical bleeding that was managed by the dentist using hemostatic sutures and localized indirect epinephrine infiltration (a constituent of the anesthetic tube primarily containing lidocaine). Consequently, the prosthesis placement was postponed. However, shortly thereafter, the patient exhibited severe hemorrhaging and was readmitted to the dental clinic. A complete prosthetic bridge was subsequently placed over the implants as a hemostatic measure to address the condition, with mild success. The patient was subsequently discharged from the clinic and instructed to commence intensive cryotherapy at home, which involved the application of multiple ice packs once every hour.

**Figure 1:**
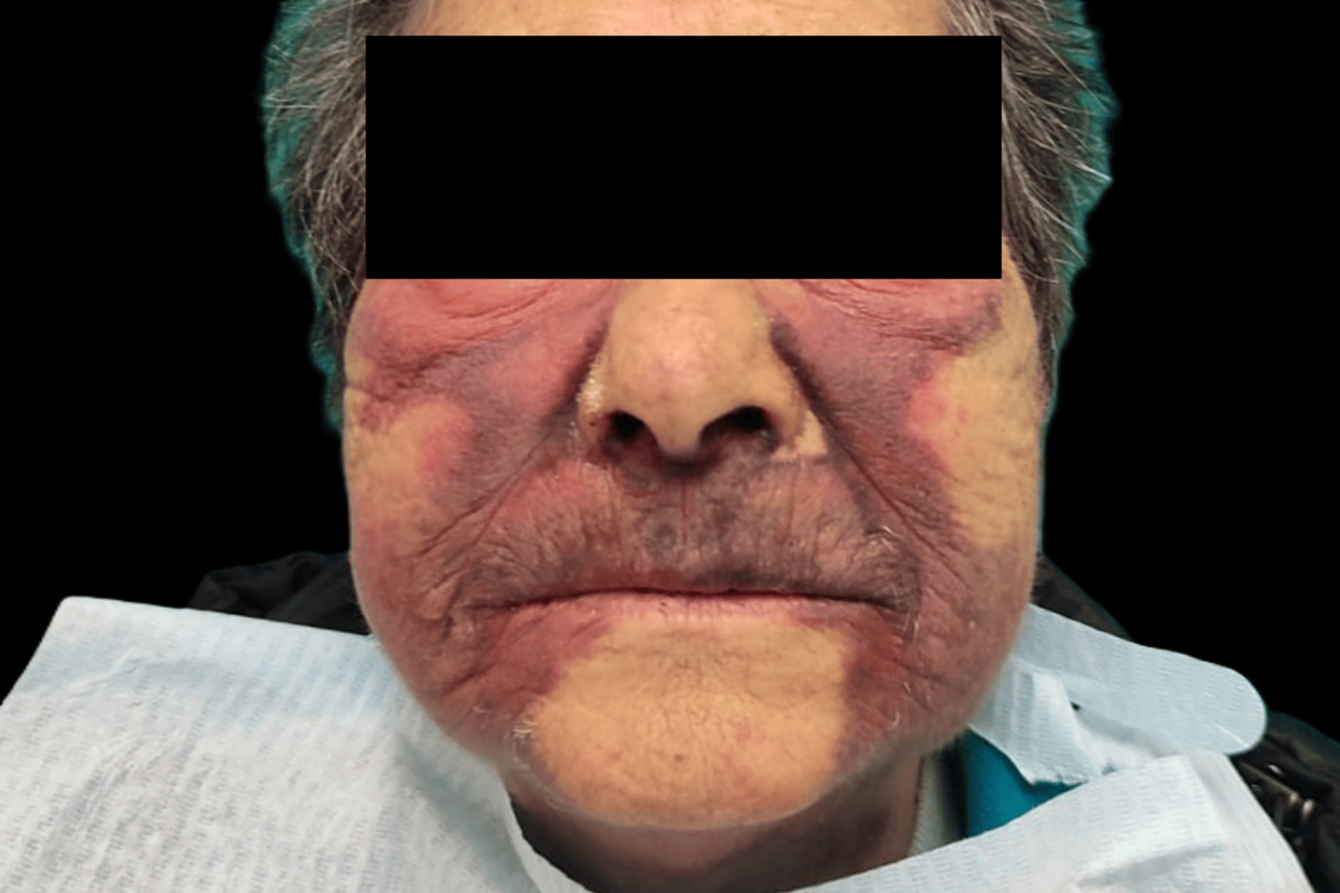
Bilateral facial bruising, extending to the cervical region

**Figure 2:**
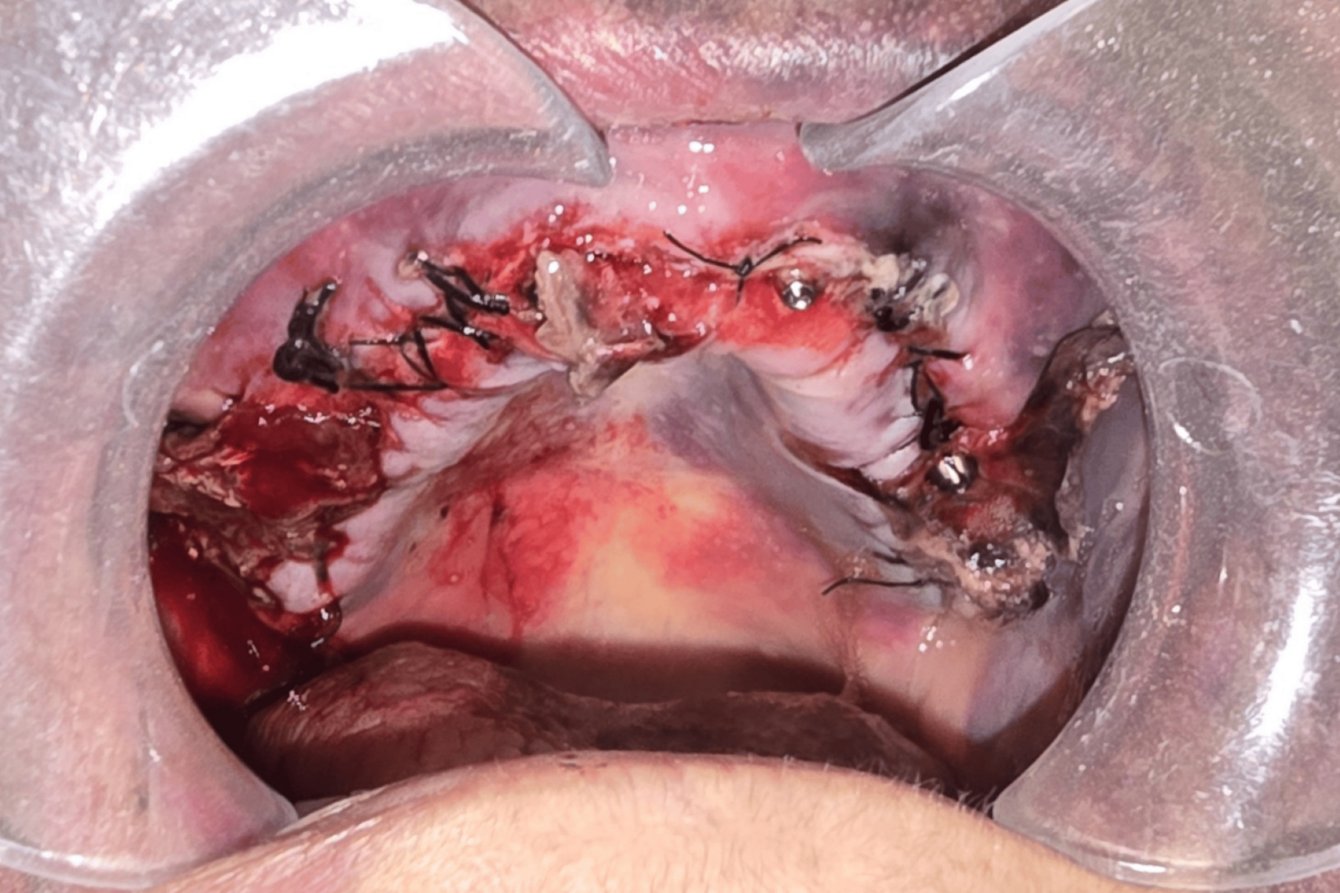
Active intraoral hemorrhage and maxillary exposure

Within 24 hours of the initial incident, the patient demonstrated significant bilateral hemifacial bruising that extended to the cervical region (Figure 1). This was accompanied by continuous maxillary bleeding and bone exposure in the first quadrant (Figure 2), with the patient returning to the dental clinic. Unable to manage the hemorrhage, the dentist opted to remove the fixed prosthesis and refer the patient to the ED.

Upon admission to the ED, laboratory findings indicated a hemoglobin level of 8.6 g/dL, which was 3 g/dL below the previously recorded value. Additionally, the INR was determined to be 1.55. The remaining components of the patient's objective examination did not reveal any additional significant alterations. The patient was hemodynamically stable, with blood pressure parameters within the optimal range.

Following the patient assessment, the original sutures were removed under local anesthesia, and a continuous locking suture technique was applied along the maxilla. Concurrently, the patient received two units of erythrocyte concentrate, resulting in a post-transfusional hemoglobin concentration of 10.1 mg/dL. Following these procedures, the patient underwent a 12-hour observation period to detect early signs of potential bleeding recurrences after which she was subsequently discharged. The patient's home prescription included amoxicillin and clavulanic acid (875/125 mg every eight hours), acetaminophen (1 g every eight hours), oral chlorhexidine digluconate 0.2% gel, and a reduction in the dosage of enoxaparin to 50 mg every 12 hours. However, less than 24 hours later, the patient returned to the ED with a new episode of active bleeding. Clinical evaluation revealed that the sutures were intact, and the bleeding was localized to the posterior region of the first maxillary quadrant and emerged between the surgical wound flaps. Given the much less exuberant presentation, a fibrin sealant (Tisseel® - Baxter International Inc., Deerfield, IL, USA) with hemostatic properties was applied and successfully stopped the bleeding.

**Figure 3:**
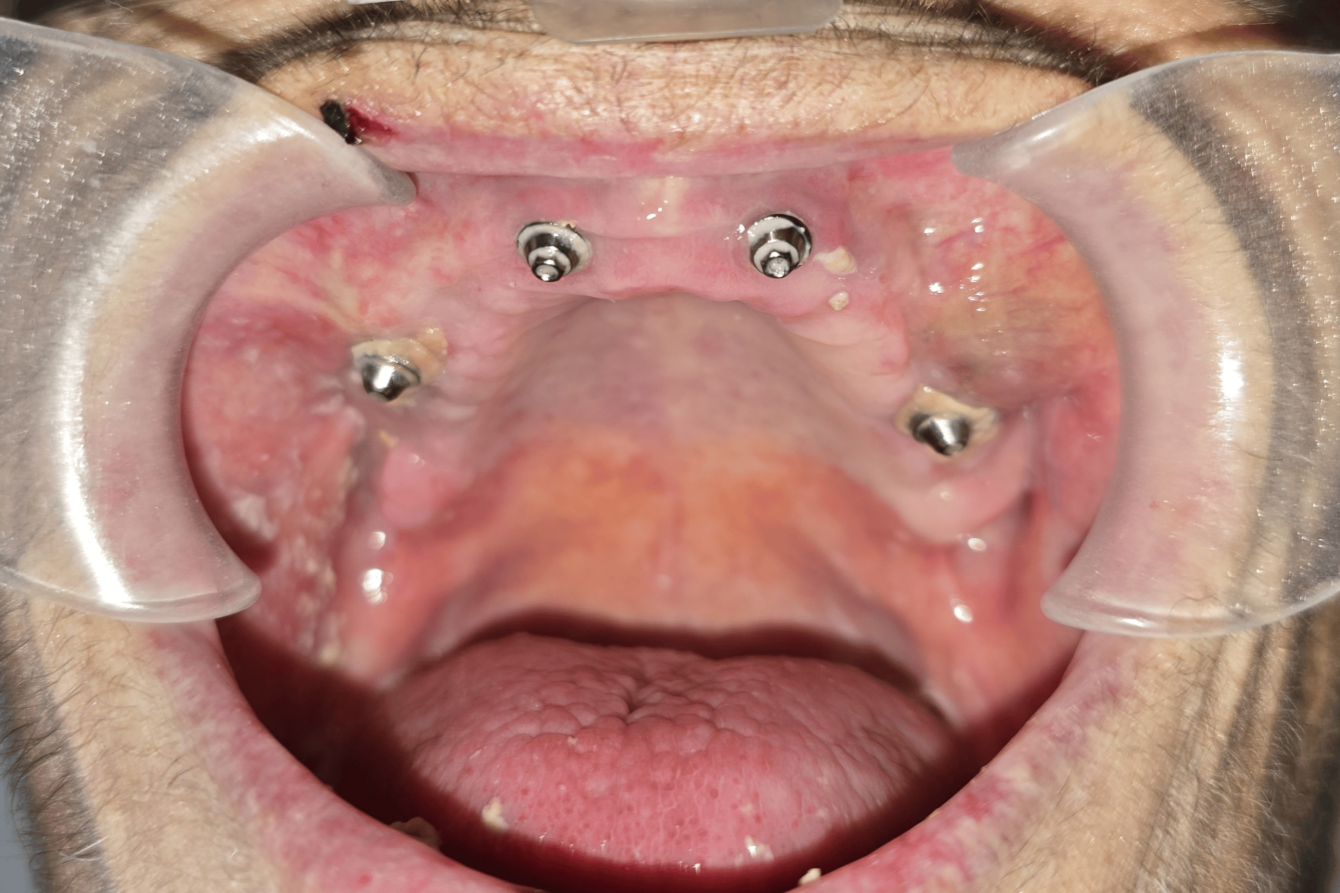
Complete healing of the maxillary wound

Patient follow-up consisted of weekly appointments until complete wound healing was achieved, which was observed after four weeks (Figure 3). During this period, strict dietary instructions were given and enforced, and the patient was restrained from reapplying the maxillary prosthesis. After being discharged from the hospital, the patient was referred back to her dental clinic, where she continued her oral rehabilitation.

## Discussion

This case illustrates the risk management involved in dental implant surgery for patients on anticoagulation therapy. It highlights the importance of meticulous preoperative assessment and patient-centered management strategies. While dental implant placement is generally considered a safe procedure, the risk of severe postoperative hemorrhagic complications remains a significant concern, especially in medically compromised patients [1,5]. According to studies in the field, the incidence of these complications can reach up to 5.7%, and patients face an elevated risk of up to 2.3 times [9,10].

Postoperative bleeding, although uncommon, can be a serious complication in oral surgery for patients on anticoagulant therapy. The risk is influenced by various factors such as the type and dosage of anticoagulants administered, the patient’s overall health, and the extent of surgical trauma [2]. In this example, switching from warfarin to enoxaparin without adjusting the dose based on the patient’s kidney function and body weight may have resulted in an increased risk of excessive bleeding. This demonstrates the importance of a thorough preoperative evaluation, including monitoring INR levels and assessing renal function, to ensure an optimal and safer surgical outcome.

Clinical guidelines advocate for a patient-centered approach to managing anticoagulation during oral surgery, prioritizing the mitigation of thromboembolic risk while reducing bleeding and its complications. The literature suggests that, in most cases, dental procedures can be performed safely without the discontinuation of anticoagulants, provided that effective hemostatic measures are implemented [3]. In this case, despite initial hemostatic measures such as suturing and indirect epinephrine infiltration, the patient experienced extensive hemorrhage requiring a more skilled intervention and complex hospital care, demonstrating the limitations of conventional techniques in high-risk patients.

Excessive bleeding in the maxillary region, as observed in this case, underlines the need for careful surgical planning and immediate intervention in such events [6,7]. The decision to remove the prosthesis to manage the hemorrhage further illustrates a clinical dilemma regarding the role of temporary prosthetic placement as a tamponade versus its potential to exacerbate bleeding due to clot disruption.

In this scenario, although the primary measures applied in the hospital were initially considered successful, the use of a fibrin sealant (Tisseel®) proved essential in controlling the bleeding, highlighting the importance of adjunctive hemostatic agents in oral surgery. Fibrin-based adhesives have proven effective in improving local hemostasis when conventional methods fail, providing additional safeguards for anticoagulated patients undergoing invasive dental procedures [8].

This patient's postoperative course underlines the critical role of meticulous observation and a well-structured postoperative plan. Frequent clinical evaluations, specific dietary limitations, and deferring prosthetic rehabilitation were imperative in averting subsequent complications and facilitating complete wound healing. These strategies mirror optimal practices for the management of high-risk surgical patients, underscoring the significance of personalized postoperative care [2,3].

## Conclusions

This case emphasizes the critical role of a multidisciplinary approach when managing patients on anticoagulation therapy who need invasive dental treatments. Developing a tailored surgical plan based on an evaluation of individual risk factors (including evaluation of kidney function and body weight, for actual dose-adjustment), combined with the careful implementation of effective hemostatic techniques, is crucial for minimizing potential complications and ensuring patient safety. By applying evidence-based guidelines and making personalized clinical decisions, healthcare professionals can optimize patient outcomes and improve the quality of care in dental implantology.
